# The effects of telehealth-delivered mindfulness meditation, cognitive therapy, and behavioral activation for chronic low back pain: a randomized clinical trial

**DOI:** 10.1186/s12916-024-03383-2

**Published:** 2024-04-12

**Authors:** Melissa A. Day, Marcia A. Ciol, M. Elena Mendoza, Jeffrey Borckardt, Dawn M. Ehde, Andrea K. Newman, Joy F. Chan, Sydney A. Drever, Janna L. Friedly, John Burns, Beverly E. Thorn, Mark P. Jensen

**Affiliations:** 1 School of Psychology, University of Queensland, 330 McElwain Building, Brisbane, QLD 4072 Australia; 2 Department of Rehabilitation Medicine, University of Washington, Seattle, WA USA; 3 Departments of Psychiatry, Anesthesia, and Stomatology, Medical University of South Carolina, Charleston, SC USA; 4 Department of Behavioral Sciences, Rush University Medical Center, Chicago, IL USA; 5 Department of Psychology, University of Alabama, Tuscaloosa, AL USA

**Keywords:** Chronic low back pain, Telehealth, Randomized clinical trial, Psychological treatment

## Abstract

**Background:**

Chronic low back pain (CLBP) is a significant problem affecting millions of people worldwide. Three widely implemented psychological techniques used for CLBP management are cognitive therapy (CT), mindfulness meditation (MM), and behavioral activation (BA). This study aimed to evaluate the relative immediate (pre- to post-treatment) and longer term (pre-treatment to 3- and 6-month follow-ups) effects of group, videoconference-delivered CT, BA, and MM for CLBP.

**Methods:**

This is a secondary analysis of a three-arm, randomized clinical trial comparing the effects of three active treatments—CT, BA, and MM—with no inert control condition. Participants were *N* = 302 adults with CLBP, who were randomized to condition. The primary outcome was pain interference, and other secondary outcomes were also examined. The primary study end-point was post-treatment. Intent-to-treat analyses were undertaken for each time point, with the means of the changes in outcomes compared among the three groups using an analysis of variance (ANOVA). Effect sizes and confidence intervals are also reported.

**Results:**

Medium-to-large effect size reductions in pain interference were found within BA, CT, and MM (*d*s from − .71 to − 1.00), with gains maintained at both follow-up time points. Effect sizes were generally small to medium for secondary outcomes for all three conditions (*d*s from − .20 to − .71). No significant between-group differences in means or changes in outcomes were found at any time point, except for change in sleep disturbance from pre- to post-treatment, improving more in BA than MM (*d* =  − .49).

**Conclusions:**

The findings from this trial, one of the largest telehealth trials of psychological treatments to date, critically determined that group, videoconference-delivered CT, BA, and MM are effective for CLBP and can be implemented in clinical practice to improve treatment access. The pattern of results demonstrated similar improvements across treatments and outcome domains, with effect sizes consistent with those observed in prior research testing in-person delivered and multi-modal psychological pain treatments. Thus, internet treatment delivery represents a tool to scale up access to evidence-based chronic pain treatments and to overcome widespread disparities in healthcare.

**Trial registration:**

Clinicaltrials.gov, NCT03687762.

**Supplementary Information:**

The online version contains supplementary material available at 10.1186/s12916-024-03383-2.

## Background

Chronic low back pain (CLBP) is a costly problem worldwide [1]. Many biomedical approaches used to treat CLBP are associated with adverse events and negative side effects [2–5]. Current treatment guidelines therefore recommend evidence-based non-invasive approaches as first-line treatment for CLBP [6].

Both in-person and internet-delivered cognitive behavioral therapy and mindfulness-based interventions have demonstrated efficacy relative to inert control conditions for managing chronic pain, including CLBP [7–10]. These treatments are sometimes combined into treatment packages (e.g., combining mindfulness-based approaches or behavioral activation with cognitive therapy) [8, 10, 11]. Three core components often included in such protocols are (1) cognitive therapy (CT) to restructure unhelpful cognitions and foster adaptive beliefs (i.e., focusing on changing *what people think*); (2) behavioral activation (BA) to reduce unhelpful behaviors and increase goal-directed behavior (i.e., focusing on changing *what people do*); and (3) mindfulness meditation (MM) to disengage from automatic habits and to enhance mindful, present moment awareness, and acceptance (i.e., focusing on *how people think*) [12–15].

Although CT and BA are key components of most integrated, multi-modal cognitive behavioral protocols, and MM is the core component of mindfulness-based interventions, to date, there have been few trials that directly compare the effects of these interventions, and the relative importance of these components and their specific effects on different outcomes are unknown. One prior study comparing behavioral therapy, CT, and a mindfulness-based stress reduction (MBSR) program found more similarities than differences in changes in a variety of outcomes [16]. However, this study did not compare core components only; one of the interventions, MBSR, contained a combination of core components. It remains possible that one or more of the core components of many psychological interventions, such as CT, BA, or MM, impact some outcomes more than others. If differential effects on specific outcomes can be identified, this knowledge could be used to improve patient-treatment matching. Further, it is not known whether treatments that focus on cognitive content, cognitive processes, or behavior result in similar effect size improvements in pain intensity and other outcomes as do more intensive, multi-modal interventions.

Given these considerations, the specific aim of the current study was to examine the relative immediate (pre- to post-treatment) and longer-term (pre-treatment to 3- and 6-month follow-up) effects of three core pain coping skills using data from a randomized clinical trial (RCT) comparing group, videoconference-delivered CT, BA, and MM for CLBP management. The primary outcome for the trial was pain interference. Secondary outcomes were pain intensity, physical function, mood (positive and negative affect), depressive symptoms, anxiety, sleep disturbance, pain medication use, and pain-related healthcare utilization. Since prior research comparing multi-component treatments has tended to find similar efficacy for different psychological pain treatments, especially for the commonly used primary outcomes of pain intensity and pain interference [10, 17–20], we hypothesized negligible effect-size between-group differences for the primary outcome of pain interference and the secondary outcome of pain intensity. We also hypothesized that the three pain interventions would produce medium effect-size improvements on both outcomes consistent with that reported for in-person delivered, multi-modal cognitive behavioral and mindfulness-based protocols [21]. Given the lack of research on comparisons between the three core interventions tested here on other outcomes, we viewed the analyses comparing their effects on the other secondary outcomes as exploratory.

## Methods

### Study design and setting

The original trial employed a three-group parallel (1:1:1), single-blind randomized clinical trial design to compare CT vs BA vs MM. We elected to not add a fourth arm to the trial comprising of an inert control condition as all three techniques have been examined in prior trials and their efficacy has been established relative to inert controls; thus, it would be unethical to assign participants to an inert control and the associated cost would be unjustifiable. This paper represents the main outcome paper related to establishing effect sizes for treatment outcome changes in the trial; however, the current analyses are considered as secondary analyses, given that the primary funded objective of the trial was to elucidate the micro- and macro-level mechanisms of action for the three treatments using ecological momentary assessment (see published protocol) [22]. All study variables were assessed remotely, and treatment was delivered in a group format via videoconferencing technology using the Zoom (https://zoom.us) platform. Study procedures were approved by the University of Washington (UW) Human Subjects Division (Identifier: STUDY00003841), and the study was pre-registered on clinicaltrials.gov (Identifier: NCT03687762) prior to commencing participant enrolment. Data collection took place between October 2018 and March 2022.

### Sample recruitment and eligibility criteria

Potential participants were identified primarily via coding lists (i.e., sections of medical charts that identify medical conditions) of UW Medicine patients who had a low back pain diagnosis in their electronic medical record. The UW Medicine setting provides a large variety of services, ranging from primary and preventative care to the most highly specialized medical care for complex conditions. Patients who did not have any clear evidence for having any of the exclusion criteria per their electronic medical record were invited to learn about the study via email and postal mailings; staff also attempted to reach each of these individuals by phone. Other recruitment strategies included the use of the UW Rehabilitation Medicine departmental research participant pool, posted flyers in pain and rehabilitation clinics, clinician referrals, news releases with the UW Newsroom, and a variety of national recruitment strategies, including use of ResearchMatch.org, social media, and posting the study on research and/or pain-related websites. Potential participants were screened for eligibility by research staff over the phone.

Study inclusion criteria were (1) ≥ 18 years of age; (2) low back pain as a primary or secondary pain condition; (3) chronic pain lasting ≥ 3 months, with pain experienced on ≥ 50% of days in the past 6 months; (4) average chronic pain intensity ≥ 3 on an 11-point scale for the past week; (5) chronic pain interference for general activities ≥ 3 on a 11-point scale for the past week; (6) able to read, speak, and understand English to comprehend the worksheets, measures, and interventions implemented; (7) if currently taking analgesic or psychotropic medication, medications must have been stabilized for ≥ 4 weeks prior to this study; (8) availability of a telephone, webcam, and microphone through computer or smartphone; and (9) access to the internet.

Study exclusion criteria were (1) headache as the primary pain condition (because the temporal nature of headache varies widely from that of most other chronic pain conditions, and the outcome measures for headache treatments differ accordingly); (2) severe cognitive impairment defined as ≥ 2 errors on the 6-Item Cognitive Screener [23]; (3) current alcohol or substance dependence [24]; (4) active malignancy (e.g., cancer not in remission), terminal illness, or serious medical condition that may interfere with either study participation or with receiving potential treatment benefits (e.g., severe lupus); (5) inability to walk at least 50 yards, which would limit the ability of participants to benefit from the BA intervention; (6) significant pain from a recent surgery or injury in the past 3 months; (7) pain condition for which surgery has been recommended and is planned; (8) any planned surgery, procedure, or hospitalization that may conflict with or otherwise influence participation in the study; (9) currently or recently receiving other psychosocial treatments for any pain condition, defined as four or more sessions within the past 12 months (as this may influence these treatment results); (10) current or past participation in a research study with treatment components that may overlap those in the current study; (11) current or history of diagnosis of primary psychotic or major thought disorder within the past 5 years; (12) psychiatric hospitalization within the past 6 months; (13) psychiatric or behavioral condition in which symptoms were unstable or severe within the past 6 months; (14) any psychiatric or behavioral issue as noted in the medical record or disclosed/observed during self-report screening that would indicate participant may be inappropriate in a group setting; and (15) a presenting symptom at the time of screening that would interfere with participation, specifically active suicidal or homicidal ideation with intent to harm oneself or others, or active delusional or psychotic thinking.

**Fig. 1:**
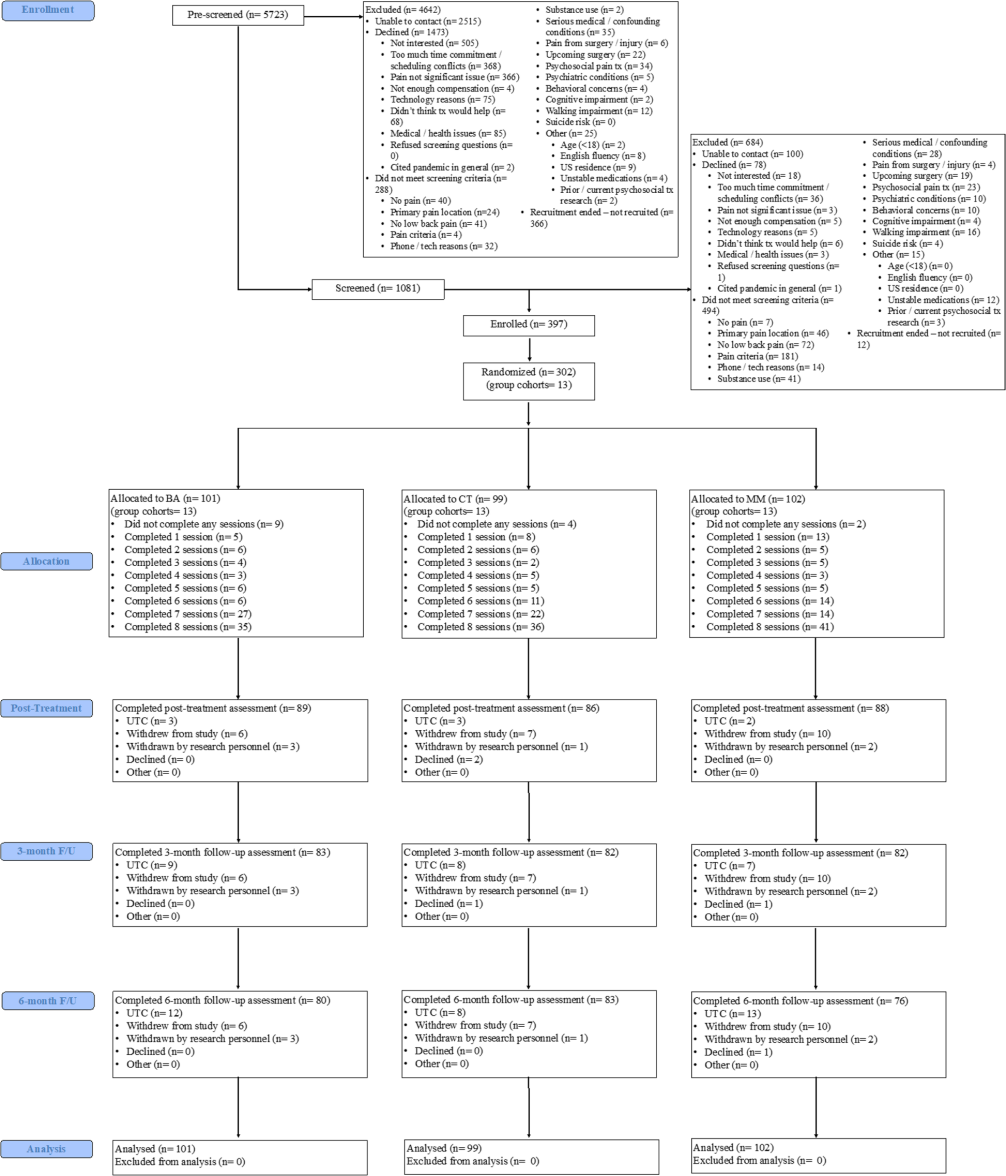
Study design and participant flow—CONSORT diagram

All participants who met eligibility criteria and provided informed consent were asked to complete the pre-treatment assessment, all baseline self-monitoring procedures, and a technology training session on the Health Insurance Portability and Accountability Act (HIPAA)-compliant (i.e., a platform with controlled authorized access and safeguards in place to protect the privacy and confidentiality of participants) Zoom videoconferencing platform used to deliver the treatment sessions (https://zoom.us). Of the 1081 participants screened for eligibility, 100 could not be contacted, 78 declined participation, 494 did not meet screening criteria, and 12 were not recruited as the recruitment period had concluded due to the target sample size having been reached. A total of *N* = 302 were randomized (*n* = 101 in BA; *n* = 99 in CT; *n* = 102 in MM). See Fig. 1 for a Consolidated Standards of Reporting Trials (CONSORT) flow diagram [25].

### Randomization

Assignment to one of the three groups was accomplished using a covariate-adaptive randomization scheme developed by the study statistician (MAC), based on the procedure described by Pocock and Simon, with the objective of balancing the covariate in the marginal distributions [26]. The covariates were biological sex, baseline pain interference score (mild/moderate or severe, as assessed via the 11-item Roland–Morris Disability Scale [27]) with cutoff for severe being a score of ≥ 7, and whether or not low back pain was the primary or a secondary pain condition.

### Concealment of treatment condition

A blinded research staff member (who was unaware of treatment allocation) communicated a numeric randomization code to an unblinded staff member, who conveyed randomization assignment and treatment schedule to participants. Participants were not informed of the study hypotheses, and all outcome assessments and data analyses were conducted by blinded research staff members who were unaware of treatment allocation. No interim data analyses were conducted.

### Study interventions

The interventions were provided simultaneously across thirteen cohorts of participants (i.e., 39 groups in all) via eight, 1.5-h Zoom-delivered group sessions delivered over 4 weeks (i.e., two sessions per week for a total of 12 h of therapy). The Zoom videoconference platform allowed participants to see and hear each other and allowed the therapist to screen-share and display visual information relevant to the respective treatment protocols (e.g., PowerPoint slides) during the sessions. The average number of participants enrolled in each group was 7.7 (standard deviation (SD) = 3.6, range = 2 to 14). The eight-session protocol length was selected as it is consistent with many prior trials and protocols (e.g., typical MBSR protocols are eight sessions long, and cognitive behavioral therapy (CBT) protocols often range from 8 to 12) [7–10]. Although in prior research, the eight sessions are typically held once per week, we elected to condense this to two sessions per week in this trial as participants were completing twice daily ecological momentary assessments (EMAs), and the participant burden of completing EMAs for 8 weeks during treatment as opposed to 4 weeks during treatment was considered excessive.

The interventions were based on standardized protocols and materials that were developed by the author team, with both the CT and MM manuals used and refined in prior trials [28–30]. The therapists were expected to follow the treatment manuals closely to ensure all content was delivered and to ensure the consistency and replicability of treatment (see fidelity monitoring procedures below). Participant workbooks specific to the respective treatment allocation were provided for participants to refer to during the group sessions, as well as additional readings and homework assignments to complete between sessions. Participants assigned to MM also received audio-recorded guided meditations to facilitate home practice.

The five therapists were post-doctoral psychology fellows or licensed psychologists with at least 2 years of clinical experience, including experience providing psychological treatments for chronic pain. They were trained and supervised to deliver all three treatments by study investigators who have considerable experience in the interventions. Therapist training, led by the investigators (MD, DE, and MJ), consisted of assigned readings [31–34] and a 6-h workshop, for each protocol (i.e., 18 h), as well as a 3-h motivational interviewing (MI) training for enhancing motivation to engage in treatment (i.e., via enhancing reflective listening practices—a MI treatment protocol was not delivered in this trial), and a 3-h training in group leadership techniques and conducting telehealth sessions, including strategies for enhancing group cohesion. Group supervision was provided (by MD, DE, and MJ) weekly during treatment delivery to support fidelity.

#### Cognitive therapy (CT)

Participants assigned to CT were taught cognitive-restructuring techniques, including how to identify automatic thoughts and recognize the connection between thoughts and feelings, behaviors, and pain [31]. Specifically, participants assigned to the CT condition were taught to (1) identify automatic thoughts related to pain, including negative or unrealistic automatic thoughts; (2) evaluate automatic thoughts for accuracy; (3) identify sources and types of distorted/unhelpful thoughts; (4) recognize the connection between automatic thoughts and emotional/physical shifts; (5) challenge negative, distorted automatic thoughts via “weighing the evidence”; (6) develop new realistic alternative cognitive appraisals; and (7) practice applying new appraisals and beliefs.

#### Behavioral activation (BA)

Participants assigned to the BA condition were given information about the negative impacts of inactivity and behavioral avoidance on chronic pain and function [33]. Participants were taught: (1) how to be aware of the activities they avoid because of fear of increasing their pain; (2) how to set effective goals so that, step by step, they can start being more active and resume some activities they enjoyed in the past but are currently avoiding; and (3) behavioral pacing skills, to facilitate an increase in appropriate activity level that is consistent with their goals. They were also encouraged to identify activity goals that were pleasurable and/or meaningful and to develop plans for achieving these activity goals.

#### Mindfulness meditation (MM)

Participants assigned to the MM condition were trained in a combination of Shamatha meditation, which involves training the mind for stability in maintaining focus on a specific object, and Vipassana, an open monitoring practice which involves acknowledging any sensory, emotional, or cognitive event that arises in the mind without evaluation, interpretation, or preference [32]. Participants were taught the following MM practices: (1) 3-min breathing space; (2) body scan meditation; (3) mindfulness of the breath and body practice; (4) mindfulness of the breath, body, sounds, and thoughts; and (5) mindfulness of the breath, body, sounds, and thoughts while also intentionally working with a difficulty through this practice. A guided inquiry of the participant’s experiences followed each in-session exercise and was also implemented in relation to discussing participants’ at-home practice.

#### Intervention fidelity

Therapist adherence to the treatment protocols and quality of treatment delivered were assessed via ratings of a random selection of 25% of session audio recordings (two randomly selected sessions per group). Ratings of both prescribed and proscribed elements were obtained. The therapists were trained by one of the principal investigators (MD) to code the therapy sessions for fidelity ratings; therapists did not rate their own sessions. Therapist adherence to treatment manuals was measured on a 0 (“none, or hardly any adherence”) to 2 (“thorough adherence”) scale, and the average adherence score was 1.98 (SD = 0.07). No prescribed treatment elements were detected in the delivery of any of the conditions. The Therapist Quality Scale, which was developed and validated in this trial, was used to rate therapist quality on a 0 (“poor quality”) to 6 (“excellent quality”) scale [35]. Higher ratings are indicative of greater quality of treatment delivery. The average therapist quality score was 4.34 (SD = 0.55).

### Measures

Readers can refer to the published protocol paper for a full list of all study measures that were administered [22]. Study data were collected and managed using Research Electronic Data Capture (REDCap) tools hosted at the Institute of Translational Health Sciences [36, 37]. The measures that were used to provide data for the analyses presented here are described below.

#### Sample characteristics

A structured pain interview was used to assess reported pain characteristics [38]. The demographic information obtained included self-reported gender, self-identified race, and self-identified ethnicity, consistent with the U.S. National Institutes of Health inclusion policies. Other participant descriptive variables assessed were years of education, relationship status, employment status, disability seeking status, and income.

### Primary outcome: pain interference

We selected five pain interference items from the Patient Reported Outcomes Measurement Information System (PROMIS) pain interference item bank to assess pain interference as the pre-specified primary outcome measure [39]. This item bank was developed using item response theory (IRT) which affords researchers the capacity to select those items from the item bank that best suit their needs. The process we used to select the five items to include in this study entailed first evaluating all of the items from the PROMIS pain interference item bank that assessed each of the key domains of pain interference described in the PROMIS interference item bank development article by Amtmann et al. [40], that is, the degree to which pain interferes with an individual’s physical, mental/emotional, and social activities. Then, using data collected in prior studies by our team and data for the original measure development sample [40], we tested three-, four-, and five-item measures with these items in order to identify the items to include, with the selection criteria being to include the fewest number of items needed (i.e., since the items were administered twice daily via EMA, which carries participant burden implications) to achieve an alpha that was > 0.80. Once the alpha for the items reached this cutoff, we stopped testing possible longer forms. Based on this process, the alpha for the five selected pain interference items, which tap a multifaceted assessment of pain interference, was excellent in the general population, 0.95 (*N* > 800) and good in the pain sample, 0.88 (*N* = 849). These five selected items ask participants to rate how much pain interferes with: (1) enjoyment of life; (2) ability to participate in leisure activities; (3) day-to-day activities; (4) social activities; and (5) household chores. Respondents rated each item on a 5-point Likert scale ranging from 1 (“Not at all”) to 5 (“Very much”), using a past 7-day recall frame. As is standard practice for PROMIS measures, the responses were computed into T-scores (i.e., mean of 50 and a standard deviation of 10 in the normative sample) [41]. The PROMIS pain interference item pool items were recommended for use by the National Institutes of Health (NIH) task force on research standards for CLBP and have substantial support for their discriminatory and prognostic importance, reliability, and construct validity [40, 42].

### Secondary outcomes

Nine secondary outcomes were assessed. Pain intensity was measured using a single-item, numerical rating scale (NRS) which asked participants to rate their average pain over the past week, on an 11-point scale ranging from 0 (“No pain”) to 10 (“Pain as bad as you can imagine”) [43–45]. The NRS has been shown to be a reliable and valid assessment of pain intensity [43–45]. To assess physical function, depressive symptoms, anxiety, and sleep disturbance, we used four-item PROMIS scales [39, 42]. As with the PROMIS pain interference items described above, each of the items assessing these domains are rated on 5-point Likert scales, and a T-score can be calculated. The scores reflect the domain as labeled; that is, higher scores represent higher levels of physical function, depression, anxiety, and sleep disturbance, respectively. The PROMIS item banks have demonstrated high reliability and construct validity [39]. The 10-item Positive and Negative Affect Schedule short-form (PANAS-SF) was used to assess general positive and negative affect. Five-items assessed positive affect and five-items assessed negative affect [46], with respondents rating the extent to which they generally feel on a 5-point scale ranging from 1 (“Never”) to 5 (“Always”). For pain medication use, participants self-reported their medication intake in the past 7 days, and the average daily morphine milligram equivalent (MME) was calculated based on published conversion factors [47]. Finally, health care utilization was assessed by asking participants to self-report on the number of visits to a health care professional in the past month.

### Adverse events

Adverse events were self-reported by participants and documented in an electronic regulatory event within REDCap. Individual adverse events were followed until the event resolved or participation in the study ended, whichever occurred first. The majority of adverse events reported by participants were conveyed to researchers without prompt (e.g., disclosed during telephone interviews with staff). Adverse event monitoring formally occurred at the beginning of each treatment session with therapists asking whether anyone had any negative effects that they attributed to the treatment practice or homework; participants with such concerns would discuss the event(s) with their therapists. Any concerns relayed to the therapist that met the definition of an adverse event were documented in REDCap by either the therapist or an unblinded staff member.

### Statistical analyses

The power analysis for the trial was based on the sample size needed to test the primary study hypotheses related to the mechanisms of these interventions, which will be reported in a planned future paper on the results of mediation models (see published protocol [22]). For the current analyses, an intention-to-treat approach was used. The primary study end-point was post-treatment (i.e., following the eight-session program, delivered over 4 weeks), and all numeric outcomes were defined as change scores computed from pre- to post-treatment (post-minus pre-treatment). The analyses also include examination of maintenance of effects, with two additional change scores computed from pre-treatment to 3-month and 6-month follow-ups. For each time point, the means of the changes in outcomes were compared among the three groups using an analysis of variance (ANOVA). The sample size varied from analysis to analysis due to missing values, ranging from 234 (at follow-up) to 298 (at the post-treatment primary end-point). The sample size for each outcome at each assessment time point is reported in the supplemental table. For each time point, we also calculated effect sizes (and 95% confidence intervals (CI)) for each intervention and between interventions for the primary outcome of pain interference, as well as for the secondary outcomes (pain intensity, physical function, depressive symptoms, anxiety symptoms, positive and negative affect, sleep disturbance). Pain medication use and pain-related healthcare utilization were categorized for each follow-up time point. Change in morphine milligram equivalent from pre-intervention to follow-up was classified as: “No prescription at pre- and follow-up,” “Increase in dose from pre- to follow-up,” “Decrease in dose from pre- to follow-up,” and “No change in dose from pre- to follow-up.” Medical services utilization was assessed by the number of health care visits in the past month and categorized as: “Decreased number from pre-treatment,” “Same number as pre-treatment,” and “Increased number from pre-treatment.” For each time point, we calculated the proportion in the category of decreased medication or decreased utilization for each intervention and between interventions. Confidence intervals of 95% were also calculated using Jeffrey’s method [48]. A comparison of distribution of the outcomes categories among the three interventions was conducted using the Pearson chi-square for homogeneity. ANOVAs were performed in SPSS v26. Effect sizes and 95% CIs were calculated in RStudio (macro *effectsize*), and proportions and Jeffrey’s 95% CI were calculated in RStudio (macro *binom*).

## Results

### Adverse events

There were 278 adverse events in the study, of which 23 were deemed to be study-related, 10 probably study-related, 3 possibly study-related, 2 unlikely study-related, and 240 not study-related. Of the 278 total adverse events, only 2 adverse events were possibly treatment related and pertained to participants in the BA group falling during an activity they sought to increase with treatment, and as a result experienced a temporary increase in pain. The other study-related adverse events were typically related to wearing the activity monitor (e.g., skin irritation with the wristband). There was a total of 27 serious adverse events in the study, none of which were study related.

### Sample characteristics

Table 1 shows demographic, social, and clinical characteristics of the sample by intervention group. There were 101 participants in the BA intervention, 99 in the CT, and 102 in the MM, with mean age of 51 (SD = 12), 47 (15), and 49 (15), respectively. Most participants were women (78.6%), White (78.2%), and with at least some higher degree of education (67.5%). Thirty-nine percent were employed and 38% were on disability, with smaller percentage being retired or having other employment status. Fifty-four percent of participants were married or living with a significant other. The average duration of pain was 15 years, with 54% reporting chronic low back pain as their primary pain source.

**Table 1:** Baseline characteristics of the study sample

Self-reported characteristic	Treatment group			Total (*N* = 302)
	Behavioral activation (*n* = 101)	Cognitive therapy (*n* = 99)	Mindfulness meditation (*n* = 102)	
**Demographics**				
Age, mean (SD)	51.0 (12.2)	46.9 (14.7)	49.4 (14.7)	49.1 (14.0)
Median (Min., Max.)	51.0 (20.0, 76.0)	46.0 (19.0, 89.0)	50.0 (19.0, 81.0)	48.5 (19.0, 89.0)
Gender identity, *n* (%)				
Woman	78 (80.4)	78 (78.8)	76 (76.8)	232 (78.6)
Man	18 (18.6)	18 (18.2)	21 (21.2)	57 (19.1)
Transgender	0 (0.0)	0 (0.0)	1 (1.0)	1 (0.3)
Non-binary	1 (1.0)	2 (2.0)	1 (1.0)	4 (1.4)
Other/unspecified	0 (0.0)	1 (1.0)	0 (0.0)	1 (0.3)
Self-identified Hispanic or Latin origin, *n* (%) yes	11 (11.2)	3 (3.0)	12 (11.9)	26 (8.7)
Self-identified race, *n* (%)				
White	75 (77.3)	83 (84.7)	72 (72.7)	230 (78.2)
Black/African American	8 (8.2)	4 (4.1)	8 (8.1)	20 (6.8)
Latinx	8 (8.2)	3 (3.1)	8 (8.1)	19 (6.5)
Other^a^	6 (6.2)	8 (8.2)	11 (11.1)	25 (8.5)
Education level, *n* (%) in category				
High school or less	4 (4.1)	9 (9.1)	10 (9.9)	23 (7.7)
Some college/technical	23 (23.5)	27 (27.3)	24 (23.8)	74 (24.8)
Associate/college degree or higher	71 (72.4)	63 (63.6)	67 (66.3)	201 (67.5)
Employment/disability status, *n* (%)				
Employed	40 (41.2)	32 (32.3)	44 (43.6)	116 (39.1)
On disability	35 (36.1)	38 (38.4)	39 (38.6)	112 (37.7)
Retired	9 (9.3)	13 (13.1)	11 (10.9)	33 (11.1)
Other^b^	13 (13.6)	16 (16.1)	7 (7.0)	36 (12.1)
Married/living with significant other, *n* (%)	54 (55.1)	52 (52.5)	55 (54.4)	161 (54.0)

^a^Includes *n* = 16 Asian; *n* = 6 American Indian/Alaska Native; *n* = 3 Native Hawaiian/Pacific Islander; also, some individuals are multiracial

^b^Includes *n* = 10 students, *n* = 10 unemployed, *n* = 6 home makers, *n* = 3 temporarily laid off, *n* = 7 other

Missing values: 4 for age, 7 for gender identity, 4 for Latinx, 8 for race, 4 for education, 5 for employment, and 4 for marital status

### Primary and secondary outcome changes

Means (SD) for raw outcomes at each time and their changes (follow-up minus pre-treatment) are shown in Additional file 1: Table S1, for every numeric outcome. Table 2 shows the effect sizes (with 95% confidence intervals) for the changes in outcomes and the results from the ANOVAs. For example, the first row shows the post- minus pre-treatment PROMIS pain interference score had a mean of − 5.6 points (SD = 5.6) for the BA group, and the effect size from pre- to post-treatment was − 1.00 (95% CI: − 1.25, − 0.73). Similar calculations are shown for the CT and MM groups. The column titled “F and P-value Group” has the F statistics and the *p*-value for the test of equality of the three means from the ANOVA. The last column of the table shows the effect sizes and their 95% CI, for comparisons of two treatments at a time. For example, when comparing BA to CT (using BA mean minus CT mean), the effect size was − 0.11 and the 95% CI was (− 0.41, 0.19). Even when an effect size is moderate or large, if the confidence interval includes zero, there is less confidence that the point estimate is likely to be real.

**Table 2:** Means, standard deviations, effect sizes, and ANOVA results of change in outcome at all time points

**Changes in outcome**	Treatment group							
	**BA**		**CT**		**MM**		*F* and *P*-value group**	Between-group effect size d***
	Mean (SD)	d* (95% CI*)*	Mean (SD)	d* (95% CI)	Mean (SD)	d* (95% CI)		
**Change in primary outcome**								
**PROMIS pain interference**								
Post-treat. minus pre-treat	− 5.6 (5.6)	− 1.00 (− 1.25, − .73)	− 5.0 (5.0)	− 1.01 (− 1.26, − .74)	− 4.5 (6.4)	− .71 (− .94, − .47)	*F*_(2,257)_ = .80 *P* = .45	BA–CT: − .11 (− .41, .19 BA–MM: − .18 (− .48, .12) MM–CT: .09 (− .21, .38)
3 months minus pre-treat	− 5.6 (6.2)	− .90 (− 1.15, − .64)	− 4.7 (5.5)	− .86 (− 1.12, − .61)	− 3.4 (6.2)	− .54 (− .78, − .31)	*F*_(2,240)_ = 2.84 *P* = .06	BA–CT: − .15 (− .46, .16) BA–MM: − .36 (− .67, .05) MM–CT: .23 (− .08, .54)
6 months minus pre-treat	− 5.6 (7.0)	− .80 (− 1.05, − .54)	− 4.1 (6.0)	− .69 (− .93, − .45)	− 3.4 (7.4)	− .47 (− .70, − .23)	*F*_(2,234)_ = 2.14 *P* = .12	BA–CT: − .23 (− .54, .08) BA–MM: − .31 (− .62, .01) MM–CT: .10 (− .21, .41)
**Change in secondary outcomes**								
**Average pain intensity**								
Post-treat. minus pre-treat	− 1.3 (1.9)	− .68 (− .91, − .44)	− 1.0 (1.8)	− .55 (− .78, − .32)	− 1.2 (2.1)	− .56 (− .78, − .33)	*F*_(2,257)_ = .51 *P* = .60	BA–CT: − .16 (− .46, .14) BA–MM: − .05 (− .35, .24) MM–CT: − .10 (− .39, .20)
3 months minus pre-treat	− 1.4 (2.2)	− .62 (− .86, − .38)	− 0.9 (1.7)	− .53 (− .76, − .29)	− 0.8 (1.9)	− .43 (− .66, − .21)	*F*_(2,242)_ = 2.10 *P* = .12	BA–CT: − .25 (− .56, .06) BA–MM: − .28 (− .59, .03) MM–CT: .04 (− .26, .35)
6 months minus pre-treat	− 1.1 (2.0)	− .54 (− .77, − .30)	− 1.0 (1.9)	− .51 (− .74, − .28)	− 1.0 (2.0)	− .52 (− .75, − .28)	*F*_(2,234)_ = .09 *P* = .91	BA–CT: − .07 (− .38, .24) BA–MM: − .03 (− .35, .28) MM–CT: .04 (− .35, .28)
**PROMIS sleep disturbance**								
Post-treat. minus pre-treat	− 4.9 (6.8)	− .71 (− .95, − .47)	− 3.5 (7.1)	− .49 (− .71, − .27)	− 1.7 (6.2)	− .27 (− .48, − .06)	*F*_(2,257)_ = 4.98 *P* = .01	BA–CT: − .19 (− .49, .11) BA–MM: − .49 (− .79, − .19) MM–CT: .28 (− .02, .57)
3 months minus pre-treat	− 4.3 (8.3)	− .52 (− .75, − .29)	-3.5 (6.6)	-.53 (-.76, -.29)	-2.1 (7.1)	-.29 (-.51, -.07)	F_(2,241)_ = 1.93 P = .15	BA—CT: -.11 (-.42, .20) BA—MM: -.29 (-.60, .02) MM—CT: .21 (-.10, .51)
6 months minus pre-treat	− 4.9 (9.4)	− .52 (− .75, − .28)	− 4.0 (7.3)	− .55 (− .78, − .31)	− 3.4 (7.6)	− .44 (− .68, − .20)	*F*_(2,234)_ = .68 *P* = .50	BA–CT: − .11 (− .41, .20) BA–MM: − .18 (− .49, .14) MM–CT: .09 (− .23, .40)
**PROMIS physical function**								
Post-treat. minus pre-treat	2.1 (3.8)	.55 (.32, .77)	1.6 (3.6)	.44 (.21, .66)	1.1 (3.4)	.32 (.11, .54)	*F*_(2,257)_ = 1.66 *P* = .19	BA–CT: .14 (− .16, .44) BA–MM: .28 (− .02, .57) MM–CT: − .14 (− .43, .16)
3 months minus pre-treat	2.7 (4.2)	.63 (.39, .87	2.0 (4.3)	.47 (.24, .70)	1.2 (4.0)	.29 (.07, .52)	*F*_(2,241)_ = 2.61 *P* = .08	BA–CT: .15 (− .16, .46) BA–MM: .36 (.05, .67) MM–CT: − .20 (− .51, .10)
6 months minus pre-treat	2.5 (4.8)	.53 (.29, .77)	1.9 (5.6)	.34 (.12, .56)	1.3 (5.2)	.25 (.02, .47)	*F*_(2,234)_ = 1.12 *P* = .33	BA–CT:.12 (− .19, .43) BA–MM: .25 (− .07, .57) MM–CT: − .12 (− .43, .20)
**PROMIS depression**								
Post-treat. minus pre-treat	− 2.6 (6.7)	− .38 (− .60, − .16)	− 3.3 (7.0)	− .46 (− .69, − .24)	− 2.6 (7.8)	− .33 (− .55, − .12)	*F*_(2,257)_ = .25 *P* = .78	BA–CT: .10 (− .20, .40) BA–MM: .01 (− .29, .31) MM–CT: .09 (− .21, .38)
3 months minus pre-treat	− 2.0 (6.4)	− .31 (− .53, − .08)	− 2.4 (6.9)	− .35 (− .57, − .12)	− 2.3 (8.2)	− .27 (− .49, − .05)	*F*_(2,241)_ = .07 *P* = .93	BA–CT: .06 (− .25, .37) BA–MM: .04 (− .27, .34) MM–CT: .02 (− .29, .33)
6 months minus pre-treat	− 1.9 (7.4)	− .25 (− .48, .03)	− 2.3 (7.7)	− .30 (− .52, − .08)	− 2.2 (9.4)	− .23 (− .46, .00)	*F*_(2,234)_ = .07 *P* = .94	BA–CT: .06 (− .28, .37) BA–MM: .04 (− .28, .35) MM–CT: .02 (− .29, .33)
**PROMIS anxiety**								
Post-treat. minus pre-treat	− 1.5 (7.2)	− .21 (− .42, .01)	− 2.2 (7.1)	− .31 (− .53, − .10)	− 2.1 (7.9)	− .27 (− .48, − .06)	*F*_(2,257)_ = .26 *P* = .78	BA–CT: .10 (− .20, .40) BA–MM: .09 (− .21, .38) MM–CT: .01 (− .28, .31)
3 months minus pre-treat	− .4 (8.3)	− .05 (− .27, .16)	− 1.4 (8.6)	− .16 (− .38, .06)	− 2.2 (9.0)	− .25 (− .46, − .02)	*F*_(2,242)_ = .84 *P* = .43	BA–CT: .11 (− .20, .042) BA–MM: .20 (− .11, .51) MM–CT: − .09 (− .40, .21)
6 months minus pre-treat	.1 (8.9)	.1 (− .22, .23)	− 1.8 (8.4)	− .22 (− .44, .00)	− 1.4 (9.4)	− .15 (− .38, .08)	*F*_(2,234)_ = 1.00 *P* = .37	BA–CT: .22 (− .09, .53) BA–MM: .16 (− .16, .48) MM—CT: .05 (-.26, .36)
**Positive affect**								
Post-treat. minus pre-treat	1.6 (3.9)	.40 (.18, .62)	1.5 (3.5)	.44 (.22, .66)	1.3 (3.0)	.44 (.22, .66)	*F*_(2,257)_ = .16 *P* = .86	BA—CT: .01 (-.29, .31) BA—MM: .08 (-.22, .38) MM—CT: -.07 (-.37, .23)
3 months minus pre-treat	1.0 (4.1)	.23 (.01, .45)	1.4 (3.6)	.39 (.16, .61)	.4 (3.4)	.13 (− .09, .35)	*F*_(2,242)_ = 1.32 *P* = .27	BA–CT: − .11 (− .42, .20) BA–MM: .14 (− .17, .44) MM–CT: − .27 (− .58, .04)
6 months minus pre-treat	.8 (4.2)	.19 (− .04, .41)	.7 (3.9)	.18 (− .03, .40)	1.1 (3.2)	.35 (.11, .58)	F_(2,234)_ = .24 P = .79	BA–CT: .02 (− .29, .33) BA–MM: − .09 (− .40, .23) MM–CT: .11 (− .20, .42)
**Negative affect**								
Post-treat. minus pre-treat	− 1.3 (3.3)	− .39 (− .61, -.17)	− 1.2 (3.2)	− .38 (− .60, − .16)	− .8 (4.1)	− .20 (− .41, .01)	*F*_(2,257)_ = .45 *P* = .64	BA–CT: − .02 (− .32, .28) BA–MM: − .13 (− .42, .17) MM–CT: .11 (− .19, .41)
3 months minus pre-treat	− 1.0 (3.0)	− .33 (− .55, − .10)	− .8 (3.4)	− .23 (− .45, − .01)	− .5 (3.6)	− .15 (− .36, .07)	*F*_(2,242)_ = .40 *P* = .68	BA–CT: − .07 (− .38, .24) BA–MM: − .14 (− .45, .17) MM–CT: .07 (− .24, .38)
6 months minus pre-treat	− 1.0 (3.9)	− .26 (− .49, − .04)	− .7 (3.8)	− .20 (− .41, .02)	− .5 (4.2)	− .11 (− .34, .12)	*F*_(2,234)_ = .38 *P* = .68	BA–CT: − .07 (− .38, .24) BA–MM: − .14 (− .45, .18) MM–CT: .07 (− 24, .38)

^*^Effect size (Cohen’s *d*) within an intervention group for a specific follow-up time point and 95% confidence interval calculated via macro *effectsize* in R for one sample

^**^F statistics and *P*-value for intervention from ANOVA with change in score as the response variable and intervention as factor

^***^Effect size (Cohen’s *d*) between intervention groups for a specific follow-up time point, and 95% Confidence Interval calculated via macro *effectsize* in R. Effect sizes shown are for the mean of first intervention minus mean of second intervention, using pooled standard deviation

In general, we observed moderate or large effects sizes within each treatment (from pre-treatment to follow-up) with decreasing or stable effects over time. Only one ANOVA showed a statistically significant result for change in outcome: change in PROMIS Sleep Disturbance from pre- to post-treatment (*F*_(2,257)_ = 4.98, *p* = 0.008), with larger changes for the BA group, followed by the CT, and with smaller changes for MM. The only 95% CI that does not include zero is for the difference between BA and MM group for change in PROMIS Sleep Disturbance from pre- to post-treatment.

Table 3 shows the distribution of categorized change in MME and healthcare utilization for each follow-up time point. For each time point, the proportion of decreased MME or utilization (which are the most important outcomes) were calculated with a 95% CI (using Jeffrey’s method). For example, the proportion of individuals who decreased MME from pre- to post-treatment in the BA group was estimated in 0.14 (95% CI = 0.08, 0.22). Similar calculations were done for the other two groups. The column title “*χ*^2^(*df*), *p*-value group” shows the chi-square statistic (with degrees of freedom) and *p*-value for the Pearson chi-square test of homogeneity (null hypothesis: same distribution in all three groups). No test was statistically significant. The last column shows the effect size for proportions (difference between two proportions) and correspondent 95% CI). All confidence intervals included 0.

**Table 3:** Medication and utilization of healthcare

Outcomes	Treatment group						*χ*^2^(*df*), *P*-value group**	Between-group difference in proportion of decreased medication/healthcare usage***
	**BA**		**CT**		MM			
	*N* (%)	Proportion (95% CI)	*N* (%)	Proportion (95% CI)	*N* (%)	Proportion (95% CI)		
**Change in average daily morphine milligram equivalent (MME) in past week**								
From pre- to post-treatment								
No prescription at pre- or post	58 (65.2)	.65 (.55, .75)	58 (68.2)	.68 (.58, .78)	61 (70.1)	.70 (.60, .79)	*χ*^2^ (6) = 10.33 *P* = .11	BA–CT: .06 (− .04, .17) BA–MM: − .01 (− .13, .10) MM–CT: .08 (− .03, .18)
Increase in dose from pre- to post	6 (6.7)	.07 (.02, .13)	9 (10.6)	.11 (.05, .18)	10 (11.5)	.12 (.06, .19)		
Decrease in dose from pre- to post	12 (13.5)	.14 (.08, .22)	6 (7.1)	.07 (.03, .15)	13 (14.9)	.15 (.09, .24)		
No change in dose from pre- to post	13 (14.6)	.15 (.08, .22)	12 (14.1)	.15 (.07, .22)	3 (3.4)	.04 (.01, .08)		
From pre- to 3 months								
No prescription at pre- or 3 months	49 (62.0)	.62 (.51, .72)	53 (65.4)	.65 (.55, .75)	53 (67.9)	.68 (.57, .78)	*χ*^2^ (6) = 4.55 *P* = .60	BA–CT: .08 (− .04, .20) BA–MM: .05 (− .08, .17) MM–CT: .03 (− .08, .14)
Increase in dose from pre- to 3 months	9 (11.4)	.12 (.05, .19)	13 (16.0)	.16 (.09, .25)	12 (15.4)	.16 (.08, .23)		
Decrease in dose from pre- to 3 months	14 (17.7)	.18 (.11, .28)	8 (9.9)	.10 (.05, .19)	10 (12.8)	.13 (.07, .22)		
No change in dose from pre- to 3 months	7 (8.9)	.09 (.04, .16)	7 (8.6)	.09 (.03, .15)	3 (3.8)	.04 (.01, .09)		
From pre- to 6 months								
No prescription at pre- or 6 months	51 (63.7)	.64 (.53, .74)	50 (64.1)	.64 (.53, .74)	48 (66.7)	.66 (.56, .77)	*χ*^2^ (6) = 6.33 *P* = .39	BA–CT: − .003 (− .11, .10) BA–MM: − .01 (− .13, .11) MM–CT: .01 (− .11, .13)
Increase in dose from pre- to 6 months	10 (12.5)	.13 (.06, .20)	11 (14.1)	.15 (.07, .22)	13 (18.1)	.18 (.10, .27)		
Decrease in dose from pre- to 6 months	10 (12.5)	.12 (.07, .22)	10 (12.8)	.13 (.07, .22)	10 (13.9)	.14 (.08, .24)		
No change in dose from pre- to 6 months	9 (11.3)	.12 (.05, .19)	7 (9.0)	.09 (.04,.16)	1 (1.4)	.02 (.00, .05)		
**Change in number of healthcare visits in past month**								
From pre- to post-treatment								
Decreased number from pre-treatment	39 (45.3)	.45 (.35, .56)	43 (50.0)	.50 (.40, .60)	38 (43.2)	.43 (.33, .54)	*χ*^2^ (4) = 2.65 *P* = .62	BA–CT: − .05 (− .21, .11) BA–MM: .02 (− .14, .18) MM–CT: − .07 (− .23, .09)
Same number as pre-treatment	24 (27.9)	.28 (.19, .38)	16 (18.6)	.19 (.11, .27)	21 (23.9)	.24 (.16, .33)		
Increased number from pre-treatment	23 (26.7)	.27 (.18, .36)	27 (31.4)	.32 (.22,.41)	29 (33.0)	.33 (.24, .43)		
From pre-treatment to 3 months								
Decreased number from pre-treatment	35 (44.3)	.44 (.34, .55)	28 (34.6)	.35 (.25, .45)	30 (36.6)	.37 (.30, .47)	*χ*^2^ (4) = 2.91 *P* = .57	BA–CT: .10 (− .07, .26) BA–MM: .08 (− .09, .24) MM–CT: .02 (− .14, .18)
Same number as pre-treatment	21 (26.6)	.27 (.17, .37)	20 (24.7)	.25 (.16, .34)	20 (24.4)	.25 (.16, .34)		
Increased number from pre-treatment	23 (29.1)	.29 (.20, .39)	33 (40.7)	.41 (.30, .51)	32 (39.0)	.39 (.29, .50)		
From pre-treatment to 6 months								
Decreased number from pre-treatment	32 (41.0)	.41 (.31, .52)	39 (47.0)	.47 (.37, .58)	32 (42.7)	.43 (.32, .54)	*χ*^2^ (4) = 1.66 *P* = .80	BA–CT: − .06 (− .23, .11) BA–MM: − .02 (− .19, .15) MM–CT: − .04 (− .21, .12)
Same number as pre-treatment	17 (21.8)	.22 (.13, .31)	19 (22.9)	.23 (.14, .32)	14 (18.7)	.19 (.11, .28)		
Increased number from pre-treatment	29 (37.2)	.37 (.27, .48)	25 (30.1)	.30 (.21, .40)	29 (38.7)	.39 (.28, .50)		

^*^Proportion of individuals who decreased medication dose or healthcare utilization and 95% confidence interval as estimated by Jeffrey’s method (Bayesian approach)

^**^χ^2^ statistics (degrees of freedom) and *P*-value from Pearson chi-square test of homogeneity (equality of distributions among categories) comparing proportion of decrease in medication or decrease in healthcare usage

^***^Difference in proportion of decrease in medication or decrease in healthcare usage (first treatment minus second treatment) and 95% confidence interval

## Discussion

This is one of the largest trials evaluating the effects of group, videoconference-delivered psychological treatments for CLBP. We found similar medium-to-large effect size improvements in the primary outcome of reduced pain interference across the three treatments studied. In general, smaller effects were found for the secondary outcomes. For all outcomes, treatment-related gains were maintained at both 3- and 6-month follow-up time points. The key study findings are consistent with prior results obtained for in-person delivered treatments evaluated in other well-powered comparison studies [16, 20, 21, 28, 49], in that more similarities than differences were found between active treatments, and observed improvements were maintained beyond the conclusion of treatment.

We did not find statistical differences between groups in means or distributions of changes in outcomes for any outcome at any time, with a single exception in change in sleep disturbance from pre- to post-treatment, which improved more in BA compared to MM. The reliability of this observed difference needs to be established in future research, given the large number of statistical tests performed here. Generally, the secondary outcomes showed small to medium effect size improvements across all three treatments, with healthcare utilization also decreasing for about 35% to 50% of the individuals (depending on time and group). Because these changes in secondary sleep- and mood-related outcomes improved without explicit therapeutic emphasis devoted to these co-occurring symptoms, this suggests they might be conceptualized as positive “side effects” of psychological pain treatment. Given the side effect profile of opioids includes symptoms such as constipation, sedation, and addiction [2], the study results provide additional support for the use of non-invasive and non-pharmacological interventions for chronic pain management as a safe and effective alternative to opioids. This is consistent with current clinical guidelines which recommend treatments such as BA, CT, and MM as first-line therapies for CLBP [6].

A further objective of the analyses conducted for this paper was to evaluate whether the core component techniques of BA, CT, and MM would result in similar changes in outcome as previously found for multimodal, combined interventions. We found that all three core components make meaningful contributions to improving pain outcomes when delivered alone, suggesting that in isolation, each technique entails active therapeutic ingredients. The overall pattern of effect sizes for the primary and secondary outcomes were found to be consistent with results obtained with combined treatment protocols, such as CBT and MBSR [16, 20, 21, 49], despite the differences in content and delivery. This indicates that all three streamlined treatments were effective and that all three can be used for pain management.

Clinically, this study has potentially important implications for addressing gaps in accessibility to evidence-based psychological pain interventions. On one level, we found that the three treatments showed similar post-treatment and follow-up effects on a wide breadth of outcomes, including on indexes of pain, mood, function, and healthcare utilization. For the sake of accessibility, these findings imply that BA, CT, and MM may be essentially interchangeable and can therefore be prescribed on the basis solely of their availability at any given location.

On a second level, the BA, CT, and MM interventions represented streamlined versions of multimodal protocols, such as CBT or MBSR. Thus, although not directly compared in this study, the interventions examined here arguably entailed less complex therapist training/skills to effectively deliver than multimodal, combined interventions (i.e., as therapists only needed to be trained to skillfully deliver one technique, such as cognitive restructuring to deliver CT, as opposed to also being skillful in delivering multiple other cognitive and behavioral techniques, as would be the case in providing CBT). This is important when considered in the context that in many countries there is currently a shortage of trained psychologists, with extensive waiting-lists [50]. This gap in healthcare is often being addressed in clinical settings by having professionals *other* than licensed psychologists now delivering psychological treatments for pain (e.g., physiotherapists). Keefe and colleagues have emphasized the importance of systematic training strategies with manuals, experiential learning, supervision, monitoring, and feedback in preparing professionals such as physiotherapists to deliver psychologically informed practice [51–54]. However, such recommendations may not have received uptake, as a recent meta-analysis of the efficacy of psychological treatments delivered by physiotherapists found only small effect sizes for pain outcome change [55]. Thus, in terms of “upskilling” professionals from other disciplines (as well as general psychologists without expertise in pain) and providing “bridging programs” for certification in the delivery of psychological techniques, it might be strategic to prioritize for such individuals to receive training in more streamlined protocols (such as the protocols investigated in this trial), as one would expect a higher quality of delivery might this way be possible.

The current findings demonstrating the meaningful benefits of these streamlined protocols also has relevance for health professionals working in inpatient settings and interdisciplinary rehabilitation settings and other fast paced environments, where research has shown that very few time-limited sessions are the most common form of psychological intervention [56]. In such brief contacts, it is typically not feasible or practical to do a “combined session” where a therapist includes both a cognitive and a behavioral technique, for example. Typically, it is recommended that one technique in a session be taught to a patient for their immediate use for coping with pain or other symptoms of concern [57, 58]. Although future research is needed to examine the translation of the current findings to such real-world settings, the results suggest that brief contact visits where a client is taught one technique (such as a cognitive restructuring technique, a mindfulness meditation practice, or a behavioral activation goal setting exercise) has the potential to result in meaningful cumulative gain.

Further related to improving access to evidence-based psychological chronic pain treatments, this trial is one of the largest undertaken to date evaluating internet, Zoom-delivered group therapy. Most prior trials testing telehealth protocols have investigated cognitive behavioral therapy [59–63], with meta-analyses concluding this delivery approach produces similar effect sizes on pain outcome improvements as found with in-person treatment [61, 62]. During the coronavirus disease (COVID-19) pandemic, there was widespread, rapid uptake of such telehealth services [64, 65], including for the treatment of chronic pain [59], to enable wider access to services. In parallel, during the pandemic an increasing number of consumers enhanced their digital literacy such that most now have the capacity to, and regularly engage in, internet-based healthcare [65]. Thus, now more than ever, internet treatment delivery represents a tool to scale up access to evidence-based chronic pain treatments. The current findings extend this body of research to indicate that BA, CT, and MM represent evidence-based protocols that can be implemented via internet and group delivery to address on-going service provision needs. The low number of treatment-related adverse events found in this study also supports the safety of Zoom delivery of the approaches investigated, and the large sample size achieved provides an indication of feasibility, interest, and need for such services.

## Limitations

There are several limitations that should be considered when interpreting the study findings. First, this trial compared three active treatments and lacked an inert (e.g., treatment as usual) and/or attention (e.g., support group) control condition. Thus, the potential effects of time (e.g., including the natural fluctuations in pain that occur over time [66]) and nonspecific factors (e.g., group cohesion, therapist attention) in contributing to the outcomes achieved cannot be determined. However, other prior trials have found treatments such as cognitive behavioral therapy and mindfulness-based stress-reduction (i.e., from which our component interventions were drawn) were more effective than inert or attention control conditions for chronic pain [8–10, 16]. In addition, as already noted, the effect sizes we found within the three interventions were comparable to those found in other studies evaluating the efficacy of psychological pain treatments and larger than the effect sizes for the control conditions in those studies [8–10, 16, 20, 21, 49]. These comparisons suggest the possibility that the benefits observed may have been related to the treatments provided. We did not actually compare these streamlined versions of CBT and MBSR to the full, multi-modal treatments, however. Thus, we can only surmise, using effect sizes from past research, the degree to which CT, MM, and BA produced similar magnitudes of effects. The effect sizes reported herein do provide guidance for future comparative efficacy studies in relation to informing sample size and power calculations. However, it is likely that such effect sizes would be moderated by various presenting baseline characteristics as well as adherence and compliance to the recommended between session homework activities, and such questions will be addressed in a planned future paper. Further, we plan to examine the EMA data collected as part of this trial (as described in the “Methods” section) to test the micro-level trajectories of change in outcomes in relation to changes in process variables. More research is needed to understand the factors that underlie why certain treatments engender meaningful benefits for some clients, but not others, as well as the optimal dose of treatment/homework for a given individual.

## Conclusions

As one of the largest telehealth trials of psychological treatments for chronic pain undertaken to date, the results suggest that a group, video-conference modality represents a safe and feasible approach to potentially improve access to chronic pain treatments for individuals globally. This is critically important for wider dissemination and telehealth implementation efforts in clinical practice, as chronic pain currently goes underdiagnosed and undertreated, with evident disparities in access to evidence-based intervention. The study findings demonstrate that isolated treatment components are associated with improvements in pain interference and other pain-related outcomes, with similar effect sizes found across the interventions. However, the similarity of outcomes across these three treatments calls into question whether the three target areas—changing cognitive content, cognitive process, and behavior—do indeed represent distinct therapeutic processes as theorized. In planned future analyses, we aim to examine both the mediators and moderators of outcome changes that may account for the improvements observed in this comparative trial to determine whether the three treatments engender benefit for the reasons specified by respective theory and for whom are they most likely to be beneficial.

## Supplementary Information

**Additional file 1:** **Table S1.** Means and standard deviations for observed outcome scores at all time points.

## Data Availability

Please contact the corresponding author with any requests for access to limited, fully deidentified data; all requests will be considered on a case-by-case basis. Because open access to data was not included as part of informed consent, access is limited.

## Abbreviations

ANOVA: Analysis of variance
BA: Behavioral activation
CLBP: Chronic low back pain
CBT: Cognitive behavioral therapy
CT: Cognitive therapy
CI: Confidence intervals
CONSORT: Consolidated Standards of Reporting Trials
COVID-19: Coronavirus disease
EMA: Ecological momentary assessment
HIPAA: Health Insurance Portability and Accountability Act
IR: Item response theory
MBSR: Mindfulness-based stress reduction
MM: Mindfulness meditation
MME: Morphine milligram equivalent
MI: Motivational interviewing
NIH: National Institutes of Health
NRS: Numerical rating scale
PROMIS: Patient Reported Outcomes Measurement Information System
PANAS-SF: Positive and Negative Affect Schedule short-form
RCT: Randomized clinical trial
REDCap: Research Electronic Data Capture
SD: Standard deviation
UW: University of Washington
