# Correction: The effects of telehealth-delivered mindfulness meditation, cognitive therapy, and behavioral activation for chronic low back pain: a randomized clinical trial

**DOI:** 10.1186/s12916-024-03693-5

**Published:** 2024-10-09

**Authors:** Melissa A. Day, Marcia A. Ciol, M. Elena Mendoza, Jeffrey Borckardt, Dawn M. Ehde, Andrea K. Newman, Joy F. Chan, Sydney A. Drever, Janna L. Friedly, John Burns, Beverly E. Thorn, Mark P. Jensen

**Affiliations:** 1 School of Psychology, University of Queensland, 330 McElwain Building, Brisbane, QLD 4072 Australia; 2 Department of Rehabilitation Medicine, University of Washington, Seattle, WA USA; 3 Departments of Psychiatry, Anesthesia, and Stomatology, Medical University of South Carolina, Charleston, SC USA; 4 Department of Behavioral Sciences, Rush University Medical Center, Chicago, IL USA; 5 Department of Psychology, University of Alabama, Tuscaloosa, AL USA

**Correction** **:** **BMC Medicine 22, 156 (2024)**

**https://doi.org/10.1186/s12916-024-03383-2**

The original article contained a sentence that lacked important clarifying information.

The sentence affected as presented in the original article is as follows in the first paragraph of the statistical analyses:

“For the current analyses, an intention-to-treat approach was used.”

The corrected sentence is as follows and supersedes the aforementioned sentence:

“For the current analyses, an intention-to-treat approach was used where every person was analyzed in their assigned treatment group, regardless of their level of compliance. Also, after considering the guidelines for the need of imputation [1–3], for each analysis, we used the data for individuals with complete data without imputation.”
