# Supplementary material for: The effects of telehealth-delivered mindfulness meditation, cognitive therapy, and behavioral activation for chronic low back pain: a randomized clinical trial
Source: BMC Med. 2024 Apr 12;22:156. doi: 10.1186/s12916-024-03383-2 (PMC11015654; doi:10.1186/s12916-024-03383-2)
Supplement: Supplementary file 1 — Additional file 1: Table S1. Means and standard deviations for observed outcome scores at all time points. [file 12916_2024_3383_MOESM1_ESM.docx]

Additional File 1, Table S1: Means and standard deviations for observed outcome scores at all time points.

| **Outcomes** | **Treatment Groups** | | | | | |
| --- | --- | --- | --- | --- | --- | --- |
|  | **BA** | | **CT** | | **MM** | |
|  | **N** | **Mean (SD)** | **N** | **Mean (SD)** | **N** | **Mean (SD)** |
| **PROMIS Pain Interference** |  |  |  |  |  |  |
| Baseline | 98 | 66.4 (5.9) | 99 | 66.6 (5.2) | 101 | 64.9 (5.4) |
| Post-intervention | 89 | 60.7 (5.1) | 86 | 61.5 (4.8) | 88 | 60.5 (6.7) |
| 3-months follow-up | 83 | 60.6 (6.4) | 81 | 61.8 (5.1) | 81 | 61.6 (6.2) |
| 6-months follow-up | 80 | 61.1 (7.2) | 83 | 62.2 (6.3) | 76 | 61.5 (7.3) |
| **Average Pain Intensity** |  |  |  |  |  |  |
| Baseline | 98 | 6.4 (1.7) | 99 | 6.6 (1.7) | 101 | 6.2 (1.4) |
| Post-intervention | 89 | 5.1 (2.0) | 86 | 5.5 (1.9) | 88 | 5.1 (2.1) |
| 3-months follow-up | 83 | 5.1 (2.1) | 82 | 5.7 (2.0) | 82 | 5.5 (2.0) |
| 6-months follow-up | 80 | 5.3 (2.1) | 83 | 5.6 (2.0) | 76 | 5.3 (2.1) |
| **PROMIS Physical Function** |  |  |  |  |  |  |
| Baseline | 98 | 36.7 (5.2) | 99 | 35.9 (4.6) | 101 | 37.7 (5.3) |
| Post-intervention | 89 | 38.7 (5.8) | 86 | 37.5 (5.8) | 88 | 38.5 (6.2) |
| 3-months follow-up | 83 | 39.3 (6.1) | 82 | 38.3 (6.1) | 81 | 38.7 (6.9) |
| 6-months follow-up | 80 | 39.2 (6.8) | 83 | 38.1 (6.2) | 76 | 39.0 (7.7) |
| **PROMIS Depression** |  |  |  |  |  |  |
| Baseline | 98 | 56.9 (8.9) | 99 | 57.7 (9.0) | 101 | 55.2 (8.6) |
| Post-intervention | 89 | 53.6 (9.1) | 86 | 54.3 (7.7) | 88 | 53.2 (9.4) |
| 3-months follow-up | 83 | 54.4 (9.5) | 82 | 54.9 (8.4) | 82 | 53.5 (9.6 |
| 6-months follow-up | 80 | 55.0 (8.9) | 83 | 54.7 (8.2) | 76 | 53.3 (10.3) |
| **PROMIS Anxiety** |  |  |  |  |  |  |
| Baseline | 98 | 57.5 (10.2) | 99 | 58.1 (8.6) | 101 | 56.7 (9.8) |
| Post-intervention | 89 | 55.2 (9.2) | 86 | 56.0 (8.4) | 88 | 55.1 (9.0) |
| 3-months follow-up | 83 | 56.4 (9.1) | 82 | 56.3 (8.3) | 82 | 54.9 (9.6) |
| 6-months follow-up | 80 | 57.2 (9.4) | 83 | 55.8 (8.8) | 76 | 55.0 (10.4) |
| **Positive Affect** |  |  |  |  |  |  |
| Baseline | 98 | 15.2 (4.3) | 99 | 15.7 (3.8) | 101 | 15.9 (3.6) |
| Post-intervention | 89 | 17.0 (4.4) | 86 | 17.3 (4.1) | 88 | 17.0 (3.4) |
| 3-months follow-up | 83 | 16.4 (4.2) | 82 | 17.4 (3.9) | 82 | 16.2 (3.5) |
| 6-months follow-up | 80 | 16.0 (4.7) | 83 | 16.7 (3.9) | 76 | 16.9 (3.8) |
| **Negative Affect** |  |  |  |  |  |  |
| Baseline | 98 | 11.8 (4.4) | 99 | 11.7 (4.2) | 101 | 10.9 (4.3) |
| Post-intervention | 89 | 10.4 (4.3) | 86 | 10.4 (3.8) | 88 | 10.1 (3.8) |
| 3-months follow-up | 83 | 10.8 (4.3) | 82 | 10.6 (3.6) | 82 | 10.4 (4.3) |
| 6-months follow-up | 80 | 10.9 (4.7) | 83 | 10.5 (3.6) | 76 | 10.1 (4.3) |
| **PROMIS Sleep Disturbance** |  |  |  |  |  |  |
| Baseline | 98 | 59.5 (7.2) | 99 | 58.9 (8.1) | 101 | 57.1 (8.4) |
| Post-intervention | 89 | 54.9 (8.4) | 86 | 55.5 (7.7) | 88 | 55.8 (7.7) |
| 3-months follow-up | 82 | 55.7 (7.5) | 82 | 55.0 (6.7) | 82 | 55.6 (9.4) |
| 6-months follow-up | 80 | 55.5 (9.2) | 83 | 52.8 (7.7) | 76 | 54.5 (10.3) |
